# Mechanism underlying the regulation of gut microbiota-metabolite axis and growth/immune function in lambs by leaf-derived polysaccharides from *Taraxacum kok-saghyz*

**DOI:** 10.3389/fvets.2026.1799716

**Published:** 2026-03-24

**Authors:** Alimu Aersilan, Zulikeyan Manafu, Gulibanu Aosiman, Xiaohui Zhang, Ziluola Tuxunjiang, Yan Meng, Saifuding Abula, Adelijiang Wusiman

**Affiliations:** 1 College of Veterinary Medicine, Xinjiang Agricultural University, Urumqi, China; 2 Xinjiang Key Laboratory of New Drug Study and Creation for Herbivorous Animal (XJ-KLNDSCHA), Xinjiang Agricultural University, Urumqi, China; 3 Crop Research Institute of Xinjiang Uygur Autonomous Region Academy of Agricultural Sciences/National Central Asian Characteristic Crop Germplasm Resources Medium-Term Gene Bank, Urumqi, China

**Keywords:** Gastrointestinal microorganisms and metabolism, growth efficiency, intestinal immunity, sheep lambs, *Taraxacum kok-saghyz* polysaccharide

## Abstract

**Introduction:**

*Taraxacum kok-saghyz* polysaccharide (TKP) possesses multiple biological activities and is a potential candidate for regulating lamb health. Using multi-omics analysis, this study investigated the immunomodulatory and growth-regulating effects of TKP produced from leaves (TKP-L) and roots (TKP-R) in lambs.

**Methods:**

Implementing untargeted metabolomics strategies and 16S rRNA gene sequencing we analyzed the mediating role of gut microbiota in improving lamb growth performance, has fully investigated how the gut microbiota-metabolite axis regulates immunological response and intestinal health.

**Results:**

The average daily gain (ADG) of lambs was considerably greater in the TKP-L group than in the control group, and the levels of Growth Hormone(GH), Insulin (INS), Insulin-like Growth Factor 1 Receptor (IGF-1R), Immunoglobulin A (IgA), Immunoglobulin G (IgG), Immunoglobulin M (IgM), and TNF-α were all significantly higher (*P* < 0.05). In the meantime, TKP-L successfully raised the expression of intestinal mucosal mucin genes and boosted the intestinal mucosa's phase of growth. TKP-L increases the relative abundances of the microbial taxa *Prevotellaceae_UCG-001, Butyrivibrio* and *Paraprevotella*, per multi-omics analysis. Metabolomics analysis indicated that TKP-L elevated the level of argininosuccinic acid by regulating the amino acid metabolism pathway. Correlation analysis showed that TKP-L altered rumen Prevotella to activate immune cell activity, also there was a significant positive relationship between the amount of argininosuccinic acid and the overall number of *Prevotellaceae_UCG-001*.

**Conclusion:**

TKP-L may significantly improve lambs' gut mucosal barrier function and growth performance. and may achieve positive regulation of lamb growth and immune function by modulating the structure of Prevotella and mediating argininosuccinic acid metabolism.

## 1 Introduction

As animal husbandry has become more intense, the risk of pathogen exposure during young livestock production continues to increase, severely constraining the healthy growth of lambs and farming efficiency (1). Factors such as environmental pathogens, poor maternal condition, and early malnutrition can all compromise the development of the lamb's immune system, reduce disease resistance, and subsequently lead to various diseases and even death (2). Currently, antibiotics, chemical drugs, and vaccines are widely used in farming. Although they safeguard animal health to a certain extent, the side effects of their overuse—such as drug residues, microbial antimicrobial resistance, and immunosuppression—are becoming increasingly prominent (3). Therefore, developing green and safe antibiotic-alternative strategies has become a vital research direction in animal husbandry. Plant-derived polysaccharides demonstrate unique potential in promoting healthy livestock farming and reducing antibiotic use due to their structural stability, excellent biocompatibility, and multi-target regulatory capabilities (4). Plant polysaccharides are not easily degraded by host enzymes; they have the capacity to specifically encourage the growth of favorable bacteria such *Lactobacillus* and Bifidobacterium, thereby improving gut microbiota structure (5). Simultaneously, ntestinal epithelial cells and immune cells can be regulated by polysaccharides and their microbial metabolites (e.g., short-chain fatty acids) via their regulatory effects (6). Studies have confirmed that dietary supplementation with polysaccharides from *Ruoqiang jujube* can improve lamb average daily gain, feed intake, feed utilization efficiency, and promote the development of body height, chest circumference, and cannon bone circumference, thereby advancing overall growth and skeletal development. Research by Zulikeyan et al. found that dietary supplementation with *Alhagi camelorum Fisch polysaccharide* in lambs effectively promoted the production of intestinal secretory immunoglobulin A (SIgA) and serum IgM. Additionally, it optimized both local intestinal and systemic immune functions in lambs by modulating the expression of cytokines linked to various T-helper cell subsets, such as TNF-α (Th0-related), IFN-γ (Th1-related), IL-4 (Th2-related), IL-17 (Th17-related), and cytokines linked to regulatory T cells (Treg) (7). According to other studies, fermented wheat bran polysaccharides can influence the composition of the rumen bacterial population in lambs, promote *Prevotella* growth, boost the manufacturing of volatile fatty acids, and aid in rumen development (8). Furthermore, dietary supplementation with sea buckthorn polysaccharides can enrich metabolic pathways related to carbohydrate metabolism, amino acid biosynthesis, and secondary metabolite biosynthesis, improving rumen fermentation efficiency and the abundance of microbial community metabolites (9). Collectively, these studies show that polysaccharide feed additives can have broad regulatory effects via the “microbiota-gut-immune” axis, effectively supporting the growth and development of young poultry and cattle while enhancing their intestinal health and immune system.

*Taraxacum kok-saghyz* Rodin, a perennial herbaceous plant of the genus Taraxacum in the Asteraceae family, possesses significant economic value in China's Xinjiang region and Central Asia due to its root's richness in natural rubber. As a congeneric species of dandelion, *Taraxacum kok-saghyz* has been traditionally used for its anti-inflammatory, anti-infective, and digestive-regulating properties (10, 11). Its root and leaf tissues are abundant in various bioactive components such as polysaccharides, flavonoids, and triterpenoids. By limiting inflammatory cell infiltration and inhibiting NLRP3 inflammasome activation, TKP has been shown to found to efficiently limit the generation of TNF-α, IL-1β, IL-6, and myeloperoxidase, thus decreasing DSS-induced oxidative stress harm (12). Current research on the bioactivity of *Taraxacum kok-saghyz* remains limited. Owing to its phylogenetic and phytochemical similarity to *Taraxacum officinale, T. kok-saghyz* is hypothesized to exhibit parallel immunomodulatory properties. Mechanistically, dandelion polysaccharides have been shown to engage Toll-like receptors (TLR4/TLR2) and complement receptor 3 (CR3), initiating MAPK/NF-κB-dependent signaling that culminates in enhanced nitric oxide (NO) synthesis and transcriptional activation of immune-related genes in RAW264.7 macrophages (13). If dandelion polysaccharides were introduced to the diet of Jian carp, the expression levels of IL-10, IL-2, HO-1, NRF2, Cu/Zn-SOD, and GPX are significantly elevated (14). Furthermore, dandelion polysaccharides demonstrate notable preventive and therapeutic effects against colitis (15). Polysaccharides extracted from dandelion root effectively mitigated weight loss, restored normal colon length, improved colonic histopathological damage (16). In a DSS-induced mouse ulcerative colitis model, it boosted the expression of intestinal tight junction proteins Occludin and ZO-1, boosted gut microbiota diversity, and increased the relative abundance of beneficial bacteria, indicating its potential use in the treatment of ulcerative colitis (17). Based on the aforementioned evidence, this study proposes the following hypothesis: as a plant of the genus *Taraxacum*, can polysaccharides extracted from *T. kok-saghyz* regulate the growth performance of lambs by modulating their gut microbiota structure and intestinal metabolite composition?

Based on this, the present investigation uses TKP as the research topic and gives it to lambs orally by gavage in order to carefully evaluate its effects on immune system function, gastrointestinal health, and growth performance. The study seeks to clarify the growth-promoting effects of the polysaccharide through tracking daily weight gain, body size indices, and the levels of growth-related hormones. Through the analysis of humoral immunity, cytokine levels, and gastrointestinal tissue morphology, it seeks to reveal its immunomodulatory mechanisms. Furthermore, by integrating gut microbiota and metabolomics analyses, the study intends to elucidate the comprehensive regulatory network through which the polysaccharide influences lamb health via “microbiota-metabolite-host” interactions. The goal of this study is to give new insights for the practice of antibiotic reduction and healthy lamb farming, as well as a theoretical basis for the use of TKP as a novel functional feed supplement.

## 2 Materials and methods

### 2.1 Preparation of TKP rodin

The preparation of TKP was performed as follows: fresh leaves and roots of *T. kok-saghyz* were collected, dried, and mixed with water at a solid-to-liquid ratio of 1:20 for two extraction cycles. After being collected, the filtrates were concentrated to a density of 1 g/ml. The concentrated solution was then subjected to ethanol precipitation using 95% ethanol to reach a final concentration of 80%, and left stationary at 4 °C for 12 h. The precipitated polysaccharides were collected and dried, yielding crude polysaccharides from TKP-L and TKP-R. The yield was determined using the phenol-sulfuric acid technique to assessing the concentration of crude polysaccharides. A Fourier-transform infrared spectrometer was utilized to identify the functional groups of TKP-L and TKP-R in the wavenumber range of 4,000–400 cm^−1^.

### 2.2 Experimental animals

Twenty-one male 75-day-old lambs with good health status and similar body weights were selected. The animal experiment was carried out at the Veterinary Hospital of Xinjiang Agricultural University. The work was authorized by Institutional Animal Care and Use Committee of Xinjiang Agricultural University (Approval No. 2025020), ensuring compliance to all pertinent ethical guidelines for animal testing and research. Six lambs from each group were randomly selected on day 28 and euthanized by exsanguination under anesthesia. Tissues from the rumen and intestines were collected for subsequent research.

### 2.3 Grouping of lambs and administration of drugs

The experimental lambs had an average body weight of 23.63 ± 0.45 kg. They were randomly divided into three groups, with seven lambs in each group: the control group (CK), the *Taraxacum kok-saghyz* leaf polysaccharide group (TKP-L), and the *Taraxacum kok-saghyz* root polysaccharide group (TKP-R). The trial period lasted 28 days. Lambs in the CK group received no additional treatment, while those in the polysaccharide groups were administered a dosage of 400 mg/kg body weight per day. All lambs had *ad libitum* access to feed and water during the experiment, and all groups were kept under the same environmental conditions, methods of management, and feeding schedule.

### 2.4 Detection of the growth performance of lambs

Body weight, height, chest circumference, and cannon bone circumference were measured at the start and finish of the trial. The average daily increase, body height, chest circumference, and cannon bone circumference were among the indicators that were computed. Additionally, blood samples were taken from the lambs' jugular veins after 28 days of eating.

### 2.5 Determination of growth factors, immunoglobulins, and cytokines in sheep

Day 28 saw the array of blood samples and filtration of serum. Enzyme-linked immunosorbent assay (ELISA) kits (Nanjing Jiancheng Bioengineering Institute, China) were used to measure the levels of GH, IGF-1 and INS. ELISA kits from Shanghai Fankewei Biotechnology Co., Ltd., China, served to evaluate serum total immunoglobulins. For the determination of intestinal immunoglobulin levels, 2 cm segments of the small intestine were homogenized in 2 ml of PBS, followed by centrifugation at 3,000 rpm for 15 min to collect the supernatant. Kits manufactured by Shanghai Fankewei Biotechnology Co., Ltd., China, served to measure the levels of serum total IgG, IgM, IgA, IL-10, IL-17, IL-21, and TNF-α.

### 2.6 Pathological examination of rumen and intestinal tissues in lambs

Tissue samples from the liver, spleen, kidneys, rumen, duodenum, jejunum, and ileum were collected on day 28 and preserved by burying these in a 4% paraformaldehyde solution. Following soaking in paraffin blocks, these tissues were cut into 3–5 μm thick slices and stained with periodic acid–Schiff (PAS) and hematoxylin and eosin (H&E). A biopsy was thereafter carried out to examine how the tissues transformed qualitatively.

### 2.7 Rumen microbial analysis

Rumen contents were collected from the lambs, transferred into sterile 15 ml centrifuge tubes, and stored at −80 °C prior to analysis. All 16S rRNA gene amplicon sequencing and downstream bioinformatic analyses were performed by Benner Biotechnology Co., Ltd. (Wuhan, China).

### 2.8 Changes in intestinal metabolites

Duodenal tissue samples were collected, placed in sterile 15 ml centrifuge tubes, and stored at −80 °C prior to analysis. Metabolite profiling and subsequent analysis of all metabolic alterations were performed by Benner Biotechnology Co., Ltd. (Wuhan, China).

### 2.9 Statistic analysis

Statistical analysis was performed using SPSS 24.0 (IBM Corp., Armonk, NY, USA) with one-way analysis of variance (ANOVA) to determine significant differences among groups. Graphical representations were generated using GraphPad Prism 5.0 (GraphPad Software, San Diego, CA, USA). All data are presented as mean ± standard error of the mean (SEM). Different superscript letters indicate statistically significant differences (*P* ≤ 0.05).

## 3 Results

### 3.1 The results of polysaccharide content and yield of leaves and roots of *Taraxacum kok-saghyz* rodin and fourier transform infrared spectroscopy analysis

**Figure 1:**
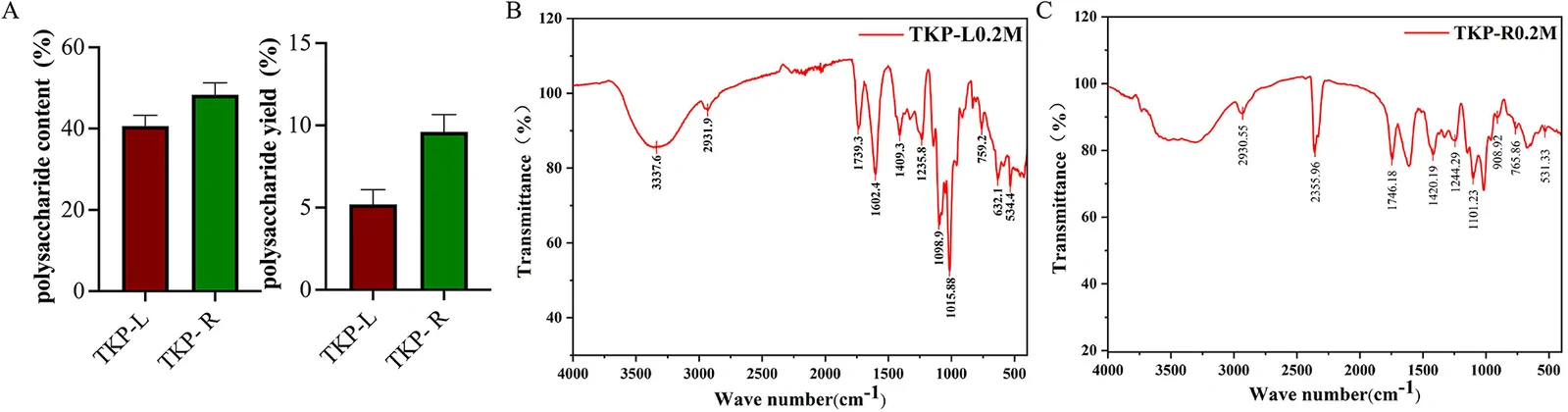
**(A)** Content and yield of TKP-L and TKP-R in crude polysaccharides. FT-IR spectrum of TKP-L **(B)** and TKP-R **(C)**. *n* = 4. Data are expressed as mean ± SD.

Crude polysaccharides were extracted from the leaves and roots of T. kok-saghyz. As shown in Figure 1A, the crude polysaccharide contents in TKP-L and TKP-R were 39.44 ± 1.98% and 48.32 ± 2.42%, respectively. The polysaccharide yields for TKP-L and TKP-R were 5.19 ± 0.77% and 9.61 ± 0.76%, respectively. The Fourier transform infrared (FT-IR) spectra of TKP-L and TKP-R showed broad absorption peaks at 3,337.6 cm^−1^ and 3,338.4 cm^−1^, respectively, as seen in Figures 1B, C. Such broad peaks are expected for the O-H stretching vibration of carbohydrates. The absorption peaks at 2,931.9 cm^−1^ for TKP-L and 2,930.5 cm^−1^ for TKP-R correspond to C-H vibrations. Peaks at 1,739.3 cm^−1^ and 1,746.18 cm^−1^ are attributed to C=O vibrations, indicating the presence of aldehyde or ester groups. Absorption peaks at 1,015.88 cm^−1^ and 1,101.2 cm^−1^ were characteristic of glycosidic linkages, which confirmed that both polysaccharides were acidic.

### 3.2 The effect of TKP on lambs' body condition and growth performance

Table 1 exhibits the variation in body traits and growth performance across the three groups of lambs. Initial body weight, initial body height, initial chest circumference, and first cannon bone circumference didn't vary substantially (*P* > 0.05) between any experimental group and the control group. The TKP-L group performed significantly better than the CK and TKP-R groups in terms of ADG and final body height on day 28 post-administration. While the TKP-R group's final body weight did not differ dramatically (*P* > 0.05) between the CK or TKP-L group, the TKP-L group's finale body weight was substantially greater than the CK group's. The average chest circumference and final cannon bone circumference of lambs in the TKP-L and TKP-R groups did not differ greatly (*P* > 0.05) with respect to the CK group.

**Table 1:** Shows that different parts of the *Taraxacum kok-saghyz* have a significant impact on the growth performance and body weight of lambs.

**Item**	**CK**	**TKP-L**	**TKP-R**	**SEM**	***P*-value**
Initial body weight (kg)	23.7	23.483	23.717	0.105	0.418
Final body weight (kg)	26.667^b^	27.633^a^	26.967^ab^	0.159	0.002
Average daily gain (g/d)	105.952^b^	148.214^a^	116.071^b^	0.006	0.001
Initial body height (cm)	52.8	52.817	52.767	0.214	0.995
Final body height (cm)	53.533^b^	55.017^a^	53.483^b^	0.262	0.026
Initial bust length (cm)	63.967	63.417	63.283	0.319	0.755
Final bust length (cm)	66.15^a^	66.983^a^	66.4^a^	0.371	0.753
Initial pipe circumference length (cm)	6.7	6.683	6.65	0.064	0.957
Final pipe circumference length (cm)	6.95^a^	7.15^a^	7.067^a^	0.058	0.136

The mean ± standard deviation (*n* = 7) is used to display the data.

A significant difference (*P* < 0.05) is shown by superscripts with distinct letters (a, b).

A non-significant difference (0.05 < *P* < 0.10) is shown by superscripts with the same letter (a, ab).

### 3.3 The effects of TKP treatment on the morphology of spleen and rumen tissues, as well as the serum biochemical and hormonal indicators of experimental animals

**Figure 2:**
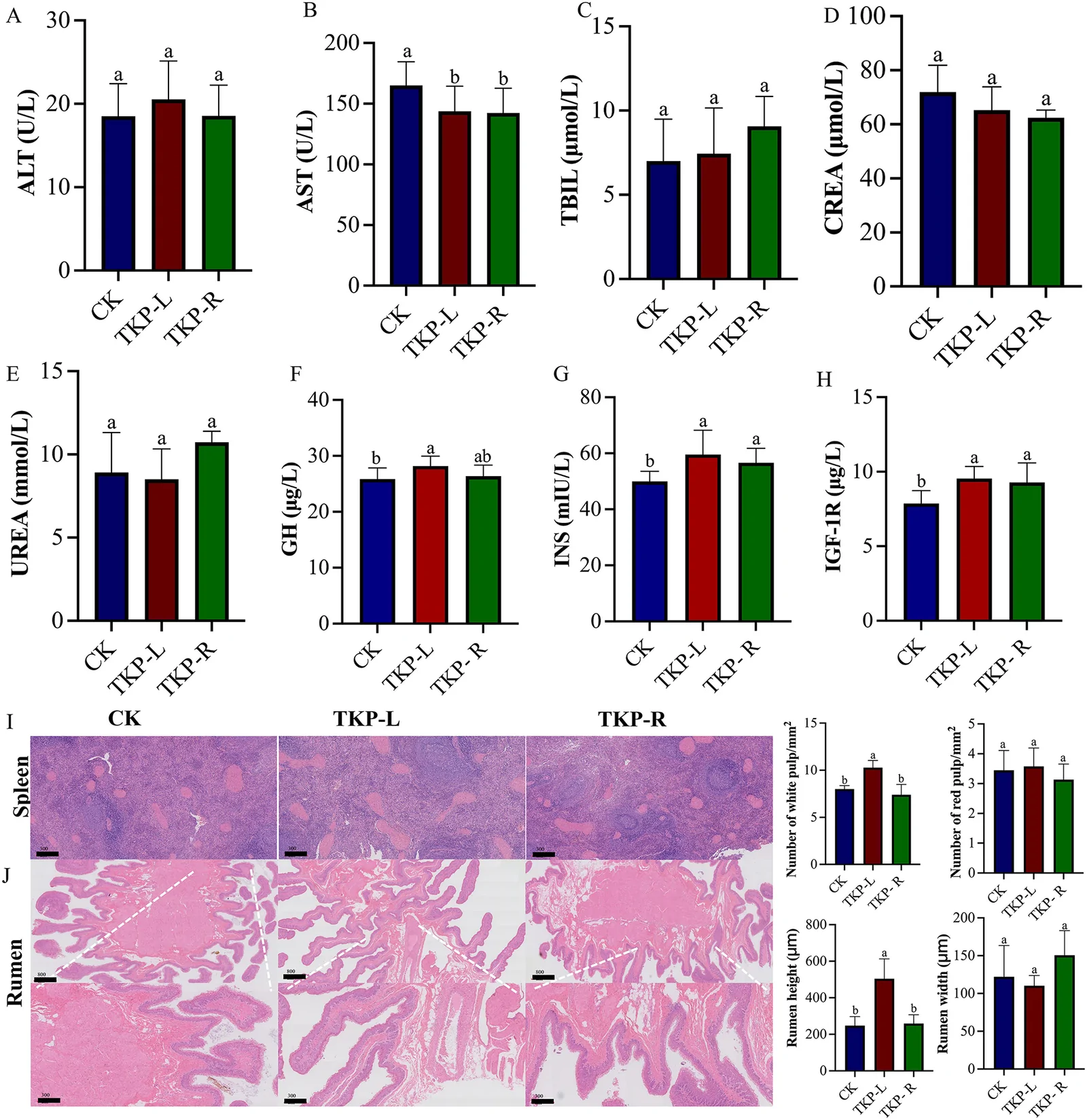
Changes in tissue and serological indicators under TKP intervention. **(A–E)** Blood biochemical analysis. The terms are as follows: aspartate transaminase (AST), alanine transaminase (ALT), total bilirubin (TBIL). **(F–H)** Sheep serum growth factors. **(I)** HE staining images of the spleen tissue and quantitative analysis of the red and white pulp of the spleen. **(J)** HE staining images of the rumen tissue and quantitative analysis of the length and width of the rumen papillae. The mean ± standard deviation (*n* = 7) is used to display the data. A significant difference (*P* < 0.05) is shown by superscripts with distinct letters (a, b); a non-significant difference (0.05 < *P* < 0.10) is indicated by superscripts with the same letter (a, ab).

To evaluate the *in vivo* safety of TKP, key serum biochemical parameters were determined in lambs of all groups. As shown in Figures 2A–E, AST, ALT, TBIL, TBA, UREA, and CREA levels were all within the normal physiological range and showed no significant differences among the treatment groups. These results indicated that TKP did not cause obvious hepatorenal toxicity or tissue damage in the lambs, confirming its good *in vivo* safety profile. Notably, Growth-related hormone analysis revealed that the TKP-L group's GH level was significantly higher than the CK group's (*P* < 0.05), however there was no significant difference between the TKP-R group and the other two groups, suggesting that TKP-L had a greater promoting effect on GH secretion (Figure 2F). There was no significant difference between the two TKP groups, though insulin (INS) levels were considerably higher in the TKP-L and TKP-R groups than in the CK group (*P* < 0.05). These results indicated that TKP effectively enhanced insulin secretion, which likely reached a near-maximal effect at the tested dosage (Figure 2G). Both TKP treatments significantly boosted insulin-like growth factor-1 (IGF-1) levels (*P* < 0.05) relative to the CK group, confirming that TKP consistently upregulates IGF-1 expression (Figure 2H). Also, there was no significant difference between the TKP-L and TKP-R groups. To investigate the effects of TKP on the lamb immune system, morphological changes in the spleen and rumen tissues were quantitatively analyzed by H&E staining to evaluate their physiological status. First, we explored whether TKP affected spleen histoarchitecture. Quantitative H&E staining findings indicated that the splenic white pulp area of the TKP-L group was substantially larger than that of the CK and TKP-R groups (*P* < 0.05), nonetheless the red pulp areas of the three groups did not vary significantly (Figure 2I). For rumen tissue, the groups' rumen papillae dimension did not differ statistically drastically. However, the rumen papillae height of the TKP-L group was far greater than that of the CK and TKP-R groups (*P* < 0.05), implying that TKP-L remedy was effectively advancing the morphological development of the ruminal mucosa (Figure 2J). In summary, TKP-L treatment significantly promoted the development of the splenic white pulp and the morphological maturation of the ruminal mucosa, with no observed toxic damage to the organism. Furthermore, TKP-L synergistically upregulated the levels of GH, INS, and IGF-1, thereby enhancing the endocrine-mediated growth regulation in lambs, demonstrating its potential application value for improving growth performance and immune development.

### 3.4 The effects of TKP treatment on the levels of serum immunoglobulins and cytokines in experimental animals

**Figure 3:**
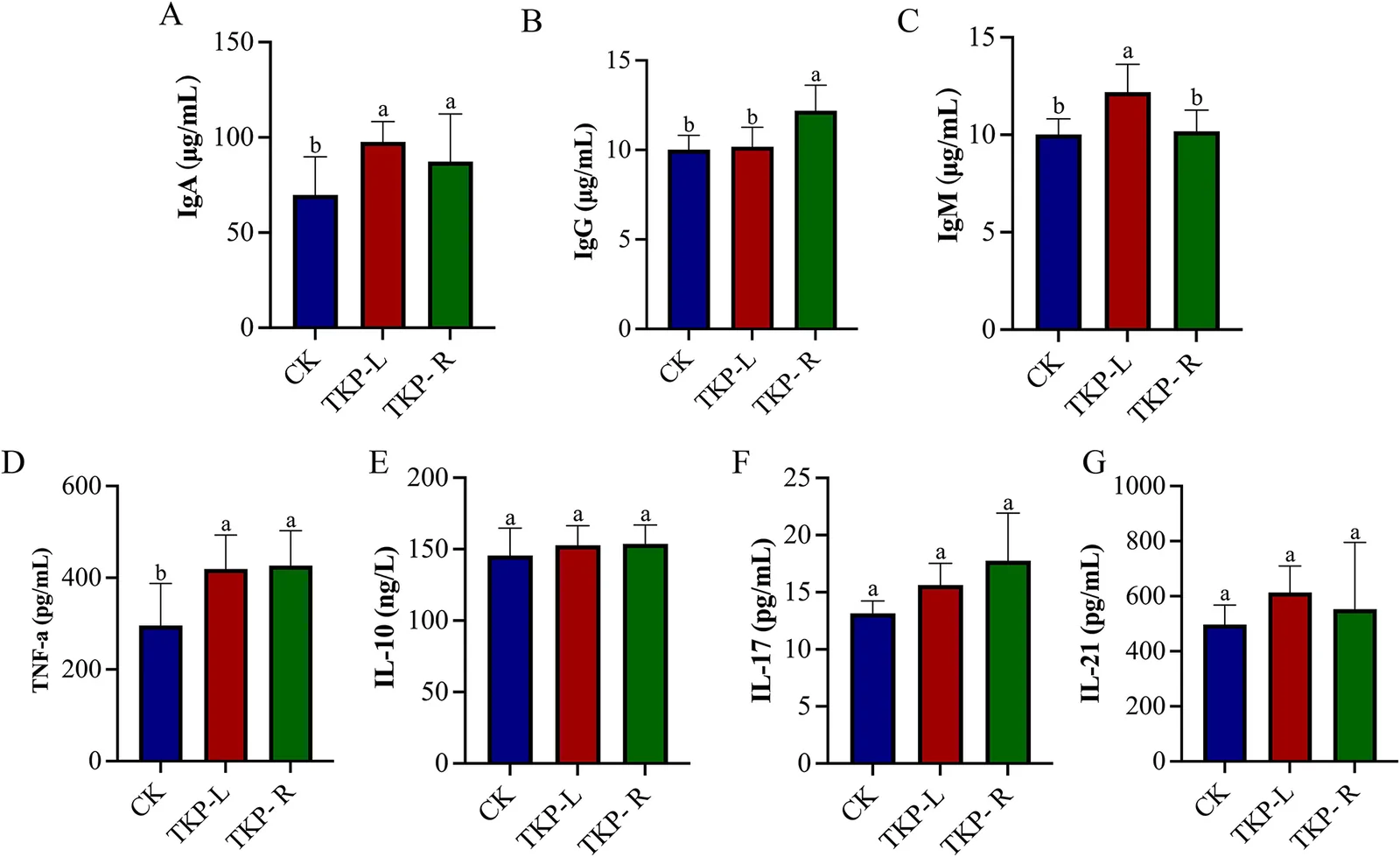
Shows the differences in immune-related indicator levels **(A–C)**, which illustrate the changes in the contents of immunoglobulins (IgA, IgG, IgM) in the three groups of samples. **(D–G)** The expression of key inflammatory factors (TNF-α, IL-10, IL-17, IL-21) is presented. The mean ± standard deviation (*n* = 7) is used to display the data. A significant difference (*P* < 0.05) is shown by superscripts with different letters (a, b).

The IgA levels in the TKP-L and TKP-R groups were considerably higher than those in the CK group (*P* < 0.05), as seen in Figure 3A, confirming that TKP greatly augmented the synthesis of antibodies pertaining to mucosal immunity. Figure 3B shows that the TKP-L group's IgG level was significantly higher than the CK group's (*P* < 0.05), indicating that TKP-L might strengthen the humoral immune response. The IgM level in the TKP-L group was dramatically higher than that in the CK group (*P* < 0.05), illustrated in Figure 3C, which implies that TKP-L drove initial generated antibodies significantly. TKP could trigger pro-inflammatory biologic responses, which is demonstrated in Figure 3D, where TNF-α levels were significantly lower in the TKP-L and TKP-R groups than in the CK group (*P* < 0.05). The IL-10 levels in the TKP-L group (about 150 ng/L) and the TKP-R group (about 155 ng/L) were both substantially larger (*P* < 0.05) than in the CK group, as shown in Figure 3E. These results suggest that TKP additionally increased the secretion of anti-inflammatory cytokines, which helped to maintain immune balance. No significant differences in IL-10, IL-17, and IL-21 levels were observed among the CK, TKP-L, and TKP-R groups (Figures 3E–G), indicating that TKP treatment had no significant effects on these immune-related cytokines.

### 3.5 The effects of different parts of TKP on the histology of sheep intestinal tissues

**Figure 4:**
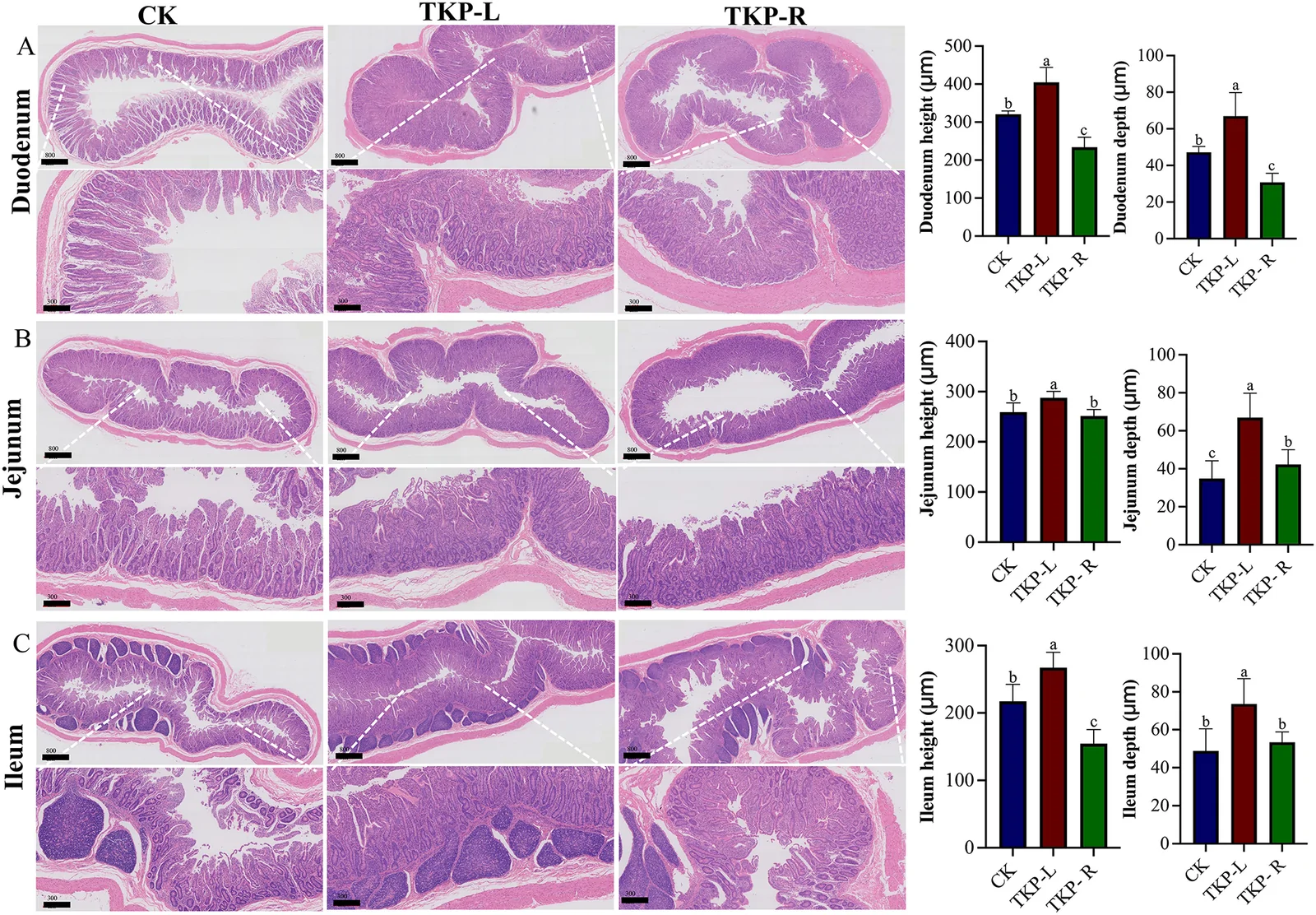
Illustrates the way AP affects sheep lambs' rumen and gut structural morphology. **(A)** Duodenal crypt depth and villus height. **(B)** The jejunum's crypt depth and villus height. Ileum's crypt depth and villus height. The data are shown as mean ± standard deviation (*n* = 5). Significant differences (*P* < 0.05) are shown by bars with various superscripts (a–c).

This study looked at how TKP treatment impacted the histological structure and morphological features (intestinal villus height and crypt depth) of intestinal tissues of experimental lambs. Figure 4A, demonstrates that the TKP-L treatment group's duodenal villus height was considerably larger than that of the CK and TKP-R groups (*P* < 0.05), and its crypt depth was significantly lower than that of the CK group (*P* < 0.05). This signifies that TKP-L successfully advanced the duodenal mucosa's developmental stage. The jejunal and ileal villus lengths in the TKP-L group were considerably larger than those in the CK and TKP-R groups (*P* < 0.05), and the crypt depths were significantly lower than those in the CK group (*P* < 0.05), as Figures 4B, C highlight. The previously stated research findings show that TKP-L can successfully enhance the intestinal mucosa's expanding phase.

### 3.6 The effect of TKP on the expression of intestinal mucin in experimental animals

**Figure 5:**
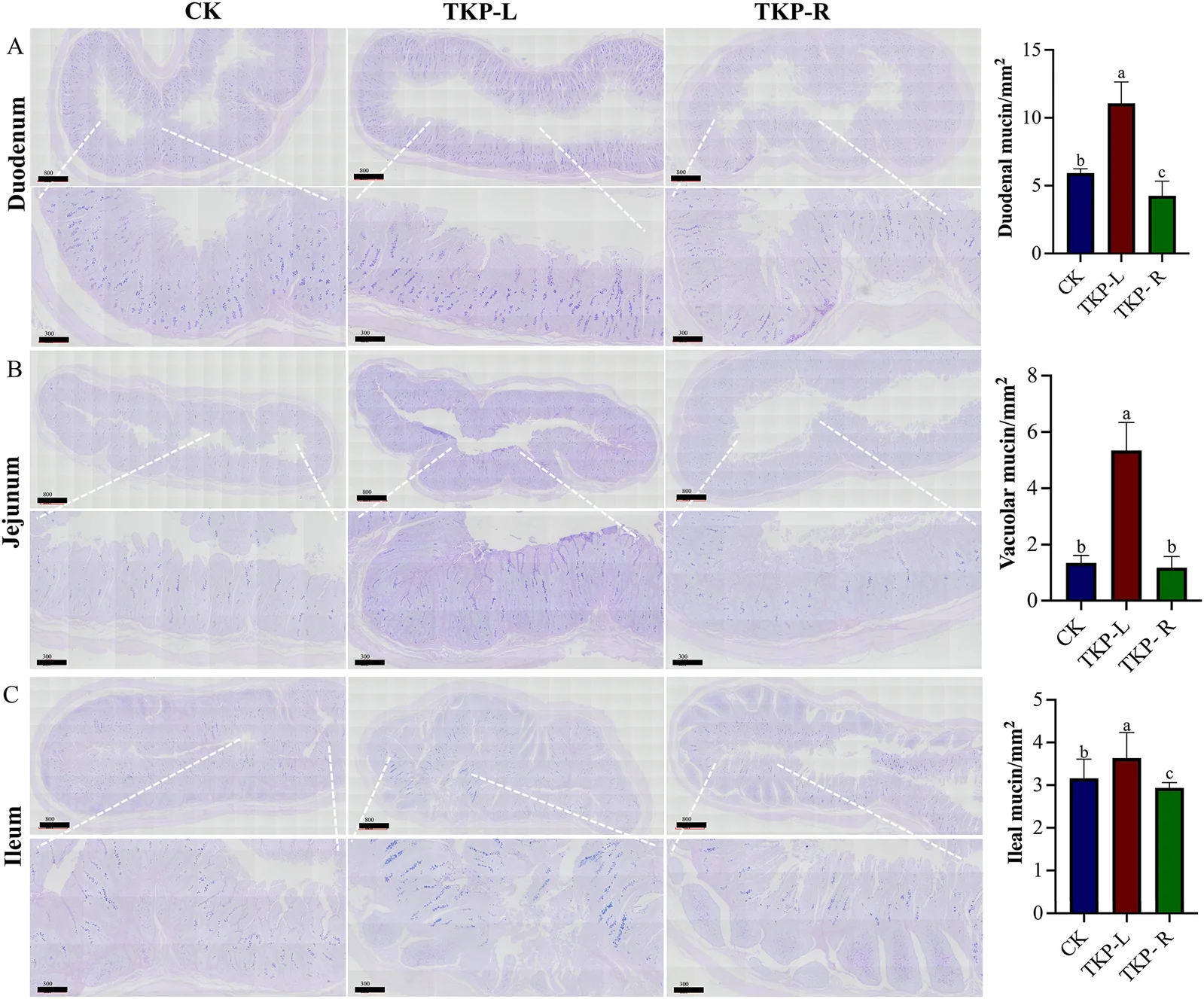
Impact of various TKP on mucin expression in sheep's ileum, jejunum, and duodenum **(A–C)**. The duodenum, jejunum, and ileum's mucosa were stained with PAS, and the amount of mucin in each intestinal segment was quantitatively assessed. The data are shown as mean ± standard deviation (*n* = 5). Significant differences (*P* < 0.05) are shown by bars with various superscripts (a–c).

Regulatory Effects of TKP Treatment on Intestinal Mucosal Mucin Expression Analyzed via PAS Staining and Quantification. The PAS staining and quantitative analysis revealed the regulatory effects of TKP treatment on the expression of intestinal mucosal mucin. The TKP-L group's mucin staining was considerably darker and covered an expanded region on the mucosal surface and inside goblet cells, as seen in Figure 5A. On the other hand, the TKP-R group's staining area and intensity were much lower, approaching closer than what was seen in the control (CK) group. Quantitatively, the TKP-L group's mucin content was much higher compared to that of the CK group (*P* < 0.05), but the TKP-R group's amount was significantly lower (*P* < 0.05). This suggests that the lower dosage of TKP (TKP-L) significantly promoted duodenal mucin secretion, whereas the higher dosage (TKP-R) exhibited an inhibitory effect. As shown in Figure 5B, mucin-positive signals in the CK and TKP-R groups were sparse and lightly stained. Mucin staining was substantially raised with dense positive signals in the TKP-L group on the surface of the intestinal villi and in the crypt goblet cells. Comparing to the CK and TKP-R groups, the TKP-L group had a significantly greater mucin content (*P* < 0.05). The CK group's mucin staining was dizzy, with positive signals mostly seen at the tips of the villi, as seen in Figure 5C. There was several deep purple positive signals apparent across the mucosal layer in the TKP-L group, which additionally demonstrated a notable increase in staining intensity and distribution range. On the opposite hand, the TKP-R group exhibited a smaller spread of positive signals and a considerable decrease in staining intensity. Quantitatively, the TKP-L group's mucin content was substantially greater than that of the CK group (*P* < 0.05), but the TKP-R group's level was significantly lower (*P* < 0.05). This suggests TKP-L can considerably boost the ileum's ability to generate mucin.

### 3.7 The influence of TKP on the rumen microbiota of lambs

**Figure 6:**
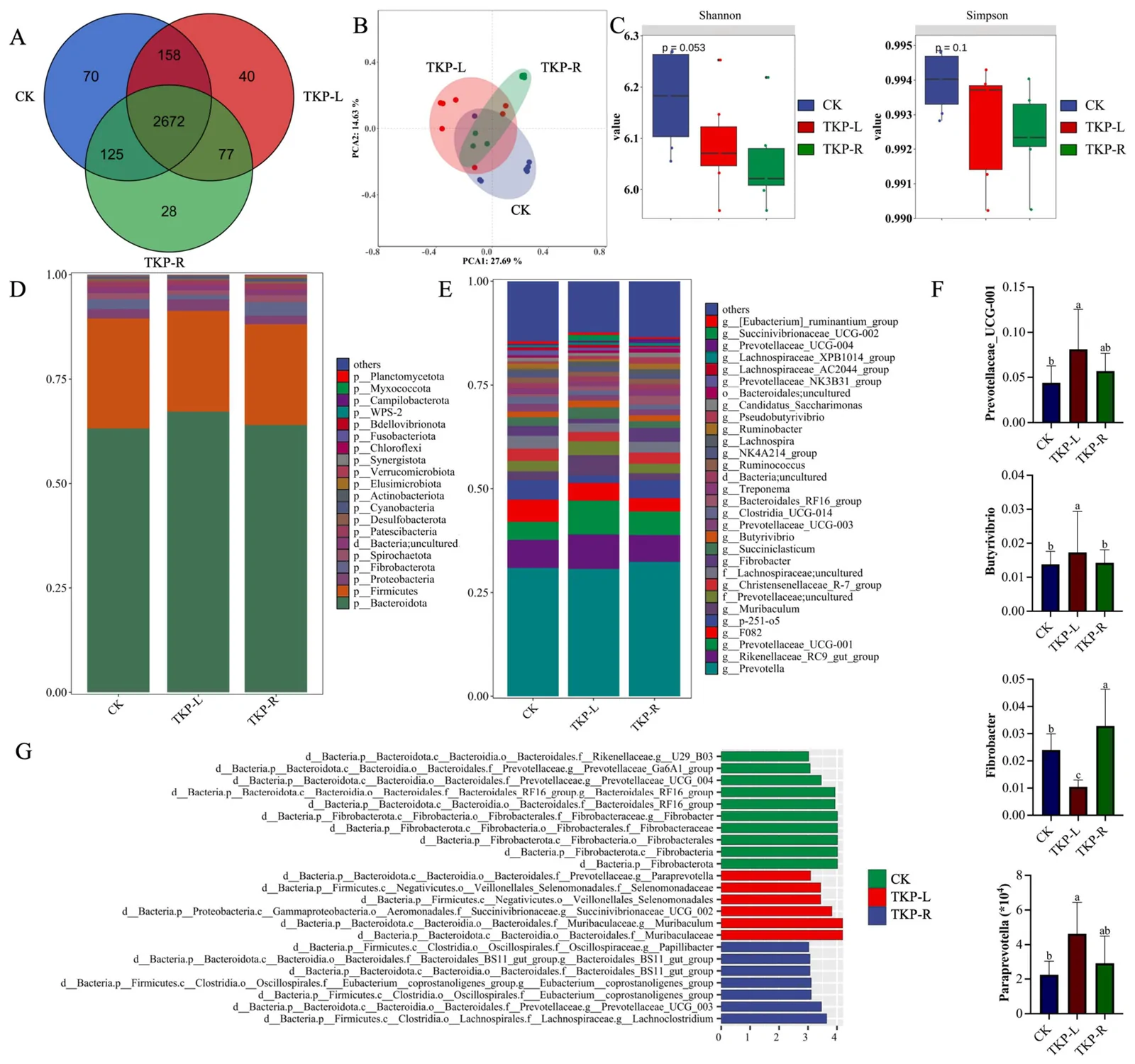
Microbial diversity of the sheep's duodenum. **(A)** Venn diagram of the distribution of amplicon sequence OTUs. **(B)** Principal coordinate analysis (PCoA) of the bacterial community in the sheep's ileum contents (based on the binary Hamming method). **(C)** α diversity of the sheep's duodenum Shannon and Simpson indices among groups. **(D, E)** Composition of the duodenum microbial flora at the phylum and genus levels. **(F)** Analysis of the total number of bacteria contributing to the differences at the phylum, class, order, family, and genus levels in the sheep's ileum using the LDA effect size (LEFSE) method. **(G)** Bar chart showing the composition of duodenum microbial species at the genus level. The mean ± standard deviation (*n* = 7) is used to display the data. A significant difference (*P* < 0.05) is shown by superscripts with distinct letters (a, b); a non-significant difference (0.05 < *P* < 0.10) is indicated by superscripts with the same letter (a, ab).

This work used 16S rRNA gene sequencing to examine the compositional features of the duodenal microbial community in order to more fully comprehend the regulatory impact of TKP on the intestinal microbiota. As to the results, 3,170 operational taxonomic units (OTUs) were discovered in all three groups, with 40 and 28 unique OTUs identified in the TKP-L and TKP-R groups, however (Figure 6A). Principal Coordinate Analysis (PCoA) revealed that axes PCoA1 and PCoA2 explained 27.69% and 14.63% of the variance in microbial community structure, respectively, showing a clear separation trend among the groups (Figure 6B). However, there was no significant differences comparing the three groups (*P* > 0.05) corresponding to the evaluation of alpha diversity indices (Shannon, Simpson) based on OTU abundance (Figure 6C). Firmicutes and Bacteroidota were both the primary phyla in the duodenal microbiota at the phylum level (Figure 6D). *Prevotellaceae_UCG-001, Butyrivibrio, Fibrobacter*, and *Paraprevotella* were among the genera whose abundances were significantly changed in the TKP-L group, based on an in-depth examination at the genus level (Figure 6E). Other results from quantitative analysis (Figure 6F): TKP-L treatment considerably reduced the relative abundance of Fibrobacter (*P* < 0.05) but significantly increased the relative abundances of *Prevotellaceae_UCG-001, Butyrivibrio*, and *Paraprevotella* (*P* < 0.05) in comparison to the CK group. On the other hand, the relative abundance of Fibrobacter was significantly increased (*P* < 0.05) by TKP-R treatment. Using Linear Discriminant Analysis Effect Size (LEfSe) analysis, differentially abundant taxa from phylum to genus level have been established among the groups of species (Figure 6G). Both *Prevotellaceae_UCG-001* and *Paraprevotella*, organisms belonging to the family *Prevotellaceae*, class *Bacteroidia*, order *Bacteroidales*, and phylum *Bacteroidota*, demonstrate significant differences in abundance between groups. As necessary genera shared by the three groups, *Prevotellaceae_UCG-001* and *Paraprevotella* are important taxa via which TKP modifies the structural makeup of the gut microbiota. TKP-L optimizes intestinal energy metabolism processes by enriching metabolism-related genera among them *Paraprevotella* and *Prevotellaceae_UCG-001*. This characteristic shift in microbiota composition is highly consistent with the regulatory effects of TKP-L on intestinal morphology, growth hormones, and immune indicators, suggesting that microbiota-mediated metabolic and immunomodulatory mechanisms are important pathways through which TKP-L exerts its growth-promoting effects.

### 3.8 Metabolites related to the duodenum

**Figure 7:**
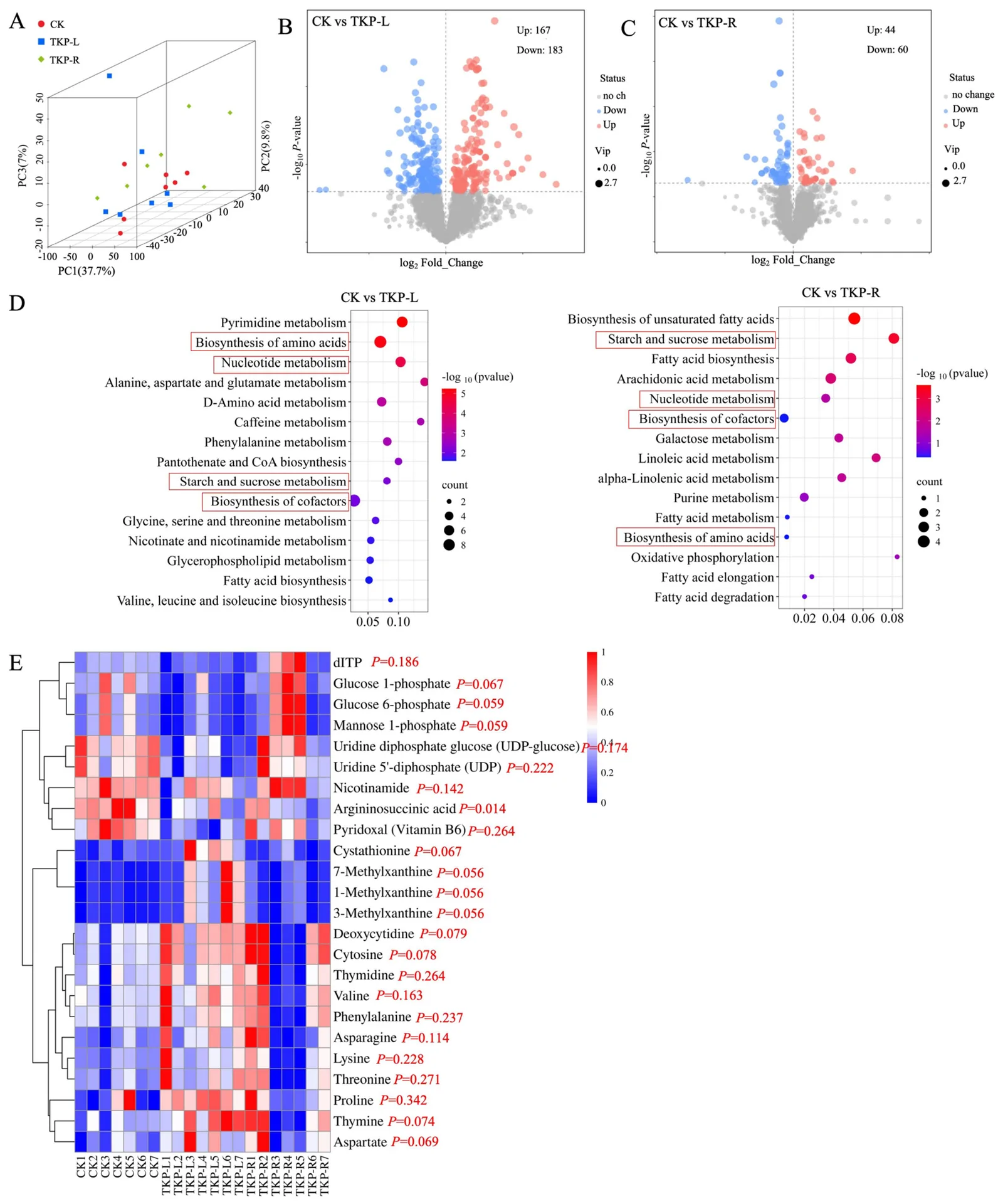
Shows how TKP therapy affected the duodenum's metabolic profile features. **(A)** Three-dimensional PCA scatter plot of duodenal metabolites; **(B, C)** Volcano plot of differential metabolites. Significantly upregulated metabolites (VIP >1, FC >1.2, *P* < 0.05) are expressed by red, significantly downregulated metabolites (VIP >1, FC < 0.8, *P* < 0.05) by blue, and metabolites with no significant difference **(D, E)** are portrayed by a bubble plot of differential metabolites enriched in KEGG pathways; the red box denotes the common core enriched pathways between the two groups; **(F)** A heatmap of differential metabolites in key metabolic pathways using hierarchical clustering. The Welch test *P*-value (*P* < 0.05 indicates significant difference) is labeled on the right side of the color gradient, which shows the standardized value of metabolite abundance (red is high abundance, blue is low). The mean ± standard deviation (*n* = 7) is used for displaying the data. A significant difference (*P* < 0.05) is shown by superscripts with distinct letters (a, b); a non-significant difference (0.05 < *P* < 0.10) is shown by superscripts with the same letter (a, ab).

To investigate the regulatory effect of TKP on the duodenal metabolite profile of lambs, this study conducted an untargeted metabolomics analysis on duodenal tissues from lambs in the CK, TKP-L, and TKP-R groups. Multivariate statistical methods were employed to construct a PLS-DA model for visual analysis. The three main components, PC1, PC2and PC3, explained 37.7, 9.8, and 7.0% of the variation, respectively, based to the primary Component Analysis (PCA) score scatter plot (Figure 7A). The metabolite profiles of duodenal tissues from the three lamb groups exhibited a significant trend of separation, indicating that TKP treatment could markedly alter the metabolic characteristics of the lambs' duodenum. Differentially abundant metabolites were eliminated using standards of VIP >1.0, FC >1.2 or FC < 0.8, and *P* < 0.05. Analysis in positive ion mode revealed: 350 differential metabolites were identified between the CK and TKP-L groups, of which 167 were significantly upregulated and 183 were significantly downregulated (Figure 7B); 104 differential metabolites were identified between the CK and TKP-R groups, with 44 significantly upregulated and 60 significantly downregulated (Figure 7C). The different metabolites from CK vs. were evaluated using KEGG pathway enrichment analysis. TKP-L and CK vs. TKP-R, The top 15 enriched metabolic pathways from both comparison groups were selected for comparative analysis (Figures 7D, E). Four pathways were found to be commonly enriched in both groups: biosynthesis of amino acids, metabolism of nucleotide metabolism of starch and sucrose, and cofactor biosynthesis. Following normalization of the metabolites within these common enriched pathways, further hierarchical clustering and differential analysis were conducted (Figure 7F). Only argininosuccinic acid demonstrated a significant difference between the groups (*P* < 0.05), according to statistical tests. These findings suggest that TKP can affect intestinal metabolic traits by controlling fundamental metabolic pathways including nucleotide and amino acid metabolism in the duodenum of lambs, with the TKP-L group showing a stronger regulatory impact. Among them, one important metabolic target that TKP-L uses to control lamb growth and development is the metabolic disruption of argininosuccinic acid.

### 3.9 The multi-omics collaborative mechanism of TKP-L in regulating intestinal function

**Figure 8:**
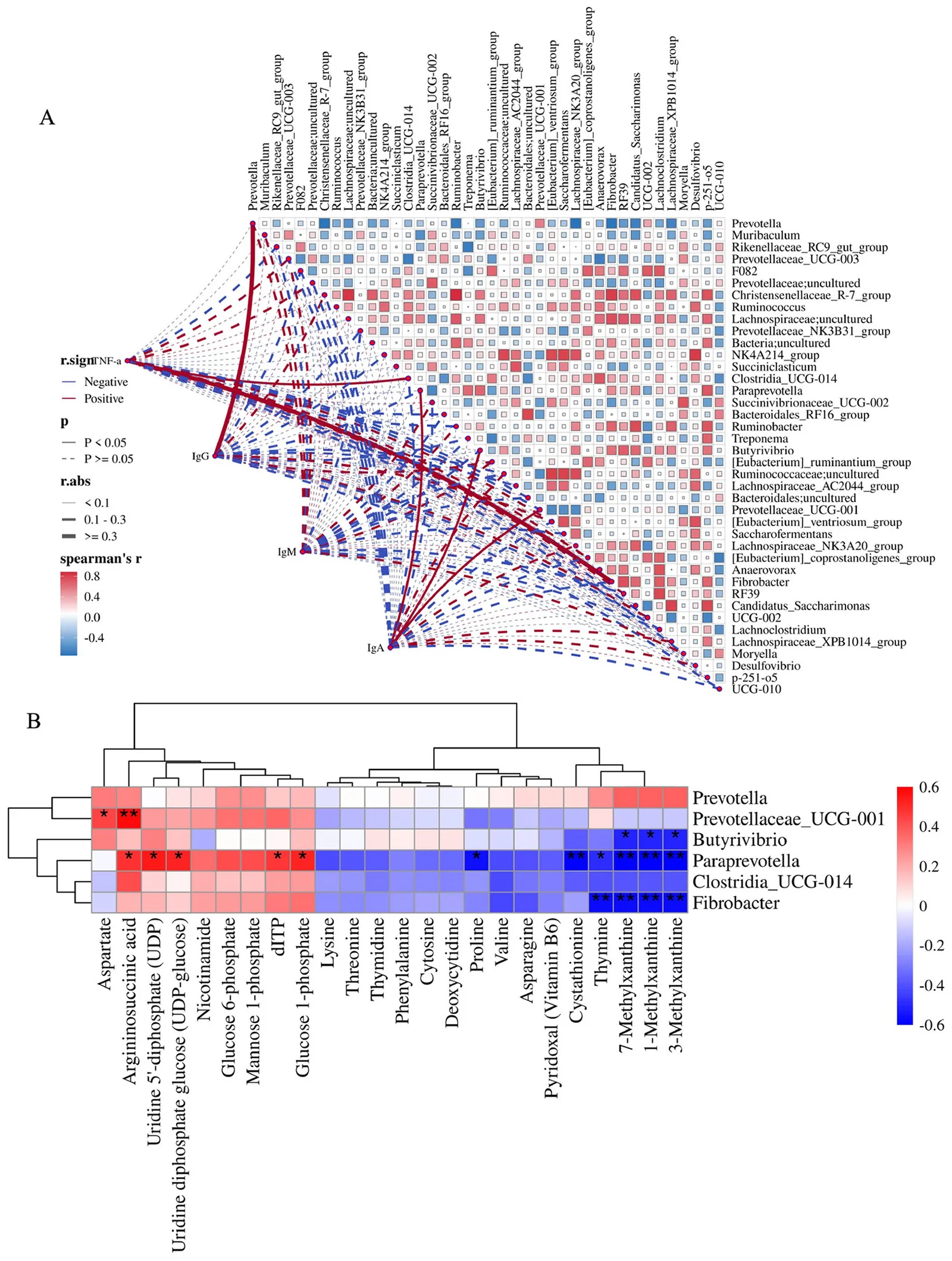
**(A)** Shows a heatmap of the correlation between serum cells and immune factors-rumen microbiota based on Spearman correlation coefficient. *P* and its corresponding line type (solid line/dotted line) indicate the statistical significance of the correlation results. The solid line represents that the correlation results have reached a statistically significant level, and the results are reliable; the dotted line represents that the correlation results have not reached a significant level, and the results may be accidental. The parameter modification module allows you to change the threshold. The correlation coefficient's absolute value, or r.abs, is indicated by the thickness of the line; the thicker the line, the greater the correlation; the thinner the line, the weaker the correlation. The parameter modification module allows you to change the threshold as well. The Pearson correlation coefficient, or Pearson r, is represented by the color of the matrix. The value of Pearson correlation coefficient r is represented by a gradient color from blue to red, where blue denotes a negative correlation, red a positive correlation, and white a weak connection. **(B)** Hierarchical clustering heatmap of microbiota and metabolites. The color gradient represents the standardized value of metabolite abundance (red indicates high abundance, blue indicates low abundance).

To elucidate the multi-omics synergistic mechanism by which TKP-L regulates intestinal function in lambs, this study first performed Spearman correlation analysis between the top 40 most abundant microbiota and serum cytokines/immunoglobulins. A metabolite-serum factor association network was constructed (Figure 8A) to systematically analyze the potential regulatory relationships between serum cytokines, immunoglobulins, and rumen microbiota perturbations. IgG showed a positive and substantial correlation with Prevotella; TNF-α demonstrated a positive and significant correlation with *Clostridia_UCG-014* and *Fibrobacter*; and IgA showed significant and beneficial interactions with the bacterial taxa *Prevotellaceae_UCG-001, Butyrivibrio*, and *Paraprevotella*. This demonstrates that TKP-L influences the activity of immune system molecules *in vivo* by modifying the rumen microbiota. Building on this, a second Spearman correlation study carried out between the metabolic compounds shared by groups in the gastrointestinal tract and the rumen microbiota that was closely linked to serum cytokines/immunoglobulins (Figure 8B). The results showed that the highly abundant intestinal *Prevotellaceae_UCG-001* exhibited a highly significant positive correlation with Argininosuccinic acid, a key metabolite in the amino acid metabolism pathway. *Prevotellaceae_UCG-001* was also significantly correlated with Aspartate, which, like Argininosuccinic acid, belongs to amino acid metabolism. *Paraprevotella* showed a significant correlation with Argininosuccinic acid, and *Paraprevotella* belongs to the same *Prevotellaceae* family as *Prevotellaceae_UCG-001*. In summary, the findings indicate that TKP-L primarily regulates the growth and development process of lambs by modulating the genus *Prevotellaceae_UCG-001* within the *Prevotellaceae* family, which mediates the metabolic process of Argininosuccinic acid in the amino acid metabolism pathway.

## 4 Discussion

A family of naturally generated polymeric macromolecules made up of aldoses or ketoses linked by glycosidic linkages, polysaccharides are widely found in organisms. In recent years, polysaccharides have garnered significant attention due to their ability to beneficially regulate the gut microbiota and their mode of action based on the “gut microbiota-metabolite” axis. As common pharmacologically active components in traditional Chinese medicine, polysaccharides are generally recognized for their potential to promote the growth of young livestock and modulate the body's immune system by improving the gut microbiota and regulating intestinal metabolites (8). With increasingly stringent global restrictions on the use of chemically synthesized feed additives (18), natural plant extracts are gradually emerging as potential alternatives to traditional growth promoters and anti-disease agents, owing to their green and safe profile, multiple biological activities, and residue-free nature (1, 19). Table 1 showed that TKP-R had a lesser growth-promoting impact, with most indicators showing just a little upward trend and not significantly distinct from the control group. However, by substantially increasing the lambs' final body weight, average daily gain, and final body height (*P* < 0.05), TKP-L efficiently promoted their growth and development.

Lambs have to possess steady liver and kidney function for themselves to grow and develop. Their functioning metabolism ensures the generation and distribution of hormones such as GH and IGF-1, as well as aiding the excretion of metabolic waste, preserving protein metabolic balance, and providing metabolic support for developing children. This is essential for safeguarding the stability of the inside environment when lambs start to develop quickly (20–22). The levels of ALT, AST, TBIL, CREA, and UREA in the TKP-treated groups were unchanged depart significantly from those in the CK group, as Figures 2A–E clearly show. These results demonstrated that TKP did not cause significant abnormalities in liver and kidney function indicators, indicating an absence of hepatorenal toxicity and good biosafety. GH, a polypeptide hormone secreted by the pituitary gland, is critical for growth and metabolic processes (23). INS regulates glucose catabolism and fat accumulation. IGF-1, a hormone that encourages cell development, proliferation, and differentiation, has an enormous impact on animal maturation (24). Figures 1F–H demonstrated that the TKP-L and TKP-R groups had far higher levels of INS and IGF-1 than the CK group (*P* < 0.05). Compared to the CK and TKP-R groups, the GH level in the TKP-L group was significantly higher (*P* < 0.05). These findings indicate the leaf polysaccharide of TKP (TKP-L) is the most efficient in increasing insulin and growth hormone release, and enhances lamb growth performance. The spleen is the largest secondary lymphoid organ and is one of the recommended organs for evaluating histopathological changes associated with immune system enhancement (Enhanced Histopathology of the Spleen). The white pulp and red pulp are the core areas for specific and non-specific immunity in the spleen. Quantitative analysis revealed an increase in white pulp in the TKP-L group (Figure 2I), suggesting that TKP-L can enhance the body's specific immune response capability against pathogens (bacteria, viruses, etc.), leading to more efficient antibody production and activation of cellular immunity. Important markers used to assess rumen and intestinal development include the length and width of the rumen papilla, the height of the small intestinal villus, and the depth of the crypt. Increasing the rumen papillae's width and length improves the rumen's capacity to soak up minerals and maintains its healthy growth (25). As shown in Figure 2J, quantitative H&E staining assessments found that the TKP-L group's rumen papilla height was considerably larger than that of the TKP-R and CK groups (*P* < 0.05), although the difference in rumen papilla breadth was not significant (*P* > 0.05). The rumen papilla width of the TKP-L group was not considerably different from that of the CK group (*P* > 0.05). This suggests that by strengthening the rumen papilla, TKP-L might increase nutrient absorption and rumen fermentation capacity.

Besides to being the main organ for digesting and absorption, the gastrointestinal tract is an essential immunological organ for shielding the body against infectious microbes (26, 27). The intestinal mucosal immunological barrier is crucial for preserving a variety of physiological processes in the gut (28, 29). By neutralizing hazardous pathogens, IgA, IgM, and IgG—the first line of antibody defense on the gastrointestinal mucosal surface—protect the delicate structure of the intestinal wall. The additional immunoglobulins in young animals' serum and their body fluids improve immune immune cells' ability to phagocytose pathogens and fight illnesses (30). Figures 3A–C demonstrate that the IgA, IgG, and IgM levels were significantly elevated in the TKP-L group in opposition to the CK group (*P* < 0.05). These results indicate that the overall upregulation of immunoglobulins suggests TKP-L can enhance both mucosal and humoral immune functions in lambs, reducing infection risk and providing stable immune protection for growth and development. IgA, IgG, and IgM output is controlled by a variety of cytokines, including IL-10, IL-17, IL-21, and TNF-α (31). Additionally, the control of the gut immune system and its protection against invasive pathogens depends on mucosal cytokines (32). TNF-α levels were considerably greater in the TKP-L and TKP-R groups than in the CK group (*P* < 0.05), as seen in Figures 3D–G. In contrast, there were no significant changes across the groups in the levels of IL-10, IL-17, and IL-21 (*P* > 0.05). These results show that by improving the body's defenses vs. infectious microbes and fostering immune protection in the lamb's gut, TKP assists to the dynamic balance of intestinal mucosal immunity.

Absorption is primarily carried out via intestinal villi. Animal growth and development are directly impacted by increased villus length, boosting the surface area readily available for nutrient absorption (33). As cell maturity and secretory function develop, the intestinal crypts—tubular glands made of epithelial cells that invade the lamina propria at the base of the villi—deepen and narrow (34). The villi in the TKP-L group were longer and more arranged, indicating more robust development, as seen in Figures 4A–C. The absence of inflammatory cell infiltration in the submucosa implies enhanced intestinal barrier function. Although the crypt depths were substantially smaller (*P* < 0.05), the TKP-L group's villus lengths in the duodenum, jejunum, and ileum were significantly longer than those of the CK group (*P* < 0.05). The research results demonstrate that TKP-L could encourage growth and development, strengthen gastrointestinal defenses, and speed up digestion and absorption through extending villus and lower crypt depth.

Mucins have functions in intestinal inflammation recuperation, gut microbiota establishment, and intestinal epidermal protection. Mucins, which are crucial for safeguarding the mucus coating on the gut surface, become synthesized through production by intestinal goblet cells (35, 36). The mucin content in the duodenum, jejunum and ileum of the TKP-L group was significantly greater than that of the CK group (*P* < 0.05), as seen in Figures 5A–C. This is consistent with the reported increase in goblet population of cells in the tissue sections. The results demonstrate that TKP-L could considerably boost mucin synthesis in the duodenum, jejunum, and ileum via accelerating the emergence of intestinal goblet cells. The intestinal mucus layer ends up greater in thickness and more connected as a result.

The gut microbiota is crucial for preserving intestinal physiology and immune function, protecting intestinal health, and improving lamb growth performance (37). Previous research showed that adding polysaccharides from traditional Chinese medicine to the diet can boost the gut microbiota's abundance in lambs (7), increasing intestinal immunity and enhancing barrier function. This leads to the idea that TKP may modulate the gut microbial ecology for it to reap its positive growth-promoting effects. This study assessed the regulatory impact of TKP on the rumen microbiota of lambs using 16S rRNA sequencing equipment. As the intake of solid feed increases in weaned lambs, the rumen microbiota tends to become more complex, with *Bacteroidota* often being the dominant phylum (38, 39) (Figure 6D), a pattern consistent with the findings of this study. *Prevotellaceae_UCG-001, Butyrivibrio, Fibrobacter*, and *Paraprevotella* were among the genera whose abundances were significantly greater in the TKP-L group compared to the CK group, according to an analysis of rumen microbiota composition and relative abundance at the genus level (Figure 6E). *Prevotellaceae_UCG-001* and *Paraprevotella* are both members of the phylum *Bacteroidota*, which has the largest relative abundance in the rumen, according to LEfSe study results. This result lines up with findings from previous investigations on lambs (40). By breaking down proteins, bacteria may help intestinal homeostasis (41, 42). Within this phylum, the genus *Prevotella*, known for protein degradation, has been shown in prior research to be positively correlated with animal growth performance indicators such as feed intake (43), feed utilization efficiency, and weight gain when it constitutes a dominant part of the gastrointestinal microbiota. This correlation matches the observed significant increase in body weight in the TKP-L group in this study (44, 45). Furthermore, studies in mice have shown that gavage with *Prevotella* can regulate the cytokine TNF-α, a conclusion consistent with the regulatory effect of TKP on cytokine levels in lambs observed in the early stages of this research (46). In summary, TKP-L can mediate the regulation of lamb growth and development by modulating the abundance and structure of Prevotella-related bacteria in the rumen. This represents a significant microbiological mechanism underlying its growth-promoting effects.

The results of the metabolomics analysis revealed that the differential metabolites in the TKP-L and TKP-R groups were enriched in a number of common pathways when compared to the CK group (Figures 7D, E). This indicates that TKP's regulatory effect on gastrointestinal metabolites in lambs may be primarily mediated through shared metabolic pathways like amino acid biosynthesis, nucleotide metabolism, starch and sucrose metabolism, and cofactor biosynthesis. Argininosuccinic acid found a significant difference between the groups (*P* < 0.05). owing to further cluster and differential analysis of metabolites within these linked pathways (47). The primary roles of Argininosuccinic acid in the body are associated with the urea cycle and amino acid metabolism (48). As a key intermediate in the urea cycle, it participates in the conversion of toxic ammonia into non-toxic urea for excretion (49). In the liver, the urea cycle is responsible for this detoxification process. Argininosuccinic acid is synthesized from citrulline and aspartate under the action of argininosuccinate synthase and is subsequently cleaved by argininosuccinate lyase into arginine and fumarate (50). This process not only aids in ammonia detoxification but also provides a pathway for arginine synthesis. In ruminants, arginine is a key amino acid that is involved in numerous biological processes, including immune regulation and protein synthesis (51). By maintaining nitrogen balance through the proper functioning of the urea cycle, Argininosuccinic acid helps regulate nitrogen metabolism homeostasis (52). As ruminants, sheep have a diet relatively high in protein, leading to increased ammonia production during metabolism. The urea cycle involving Argininosuccinic acid ensures timely conversion of ammonia to urea for excretion, preventing ammonia accumulation and potential toxicity, thereby maintaining the body's nitrogen balance and internal environment stability. Furthermore, arginine produced from the cleavage of Argininosuccinic acid serves as a crucial substrate for multiple physiological processes. It participates in nitric oxide synthesis, which plays vital roles in vasodilation, immune defense, and neurotransmission (53). In conclusion, TKP-L can mediate the metabolism of Argininosuccinic acid by regulating core metabolic pathways such as amino acid biosynthesis, thereby providing essential metabolic support for protein metabolism, immune regulation, and the growth and development of lambs.

A Spearman correlation study was carried out between circulating immune cells/immune factors and highly prevalent genera in the rumen microbiota based on integrated multi-omics data. The results showed that Paraprevotella and *Prevotellaceae_UCG-001*, both belonging to the *Prevotellaceae* family, exhibited significantly positive correlations with IgG and IgA (Figure 8A). This validates the core hypothesis of this trial, indicating that TKP-L can modulate the expression of host immune factors by regulating the rumen microbiota. *In vitro* experiments by Jiatai Gong, Siqi Ma, et al. have confirmed that argininosuccinic acid is a key metabolite of the *Prevotella* genus, which falls in line with the results of the trial's correlation research between gut microbiota and metabolomics (54) (Figure 8B). In summary, these findings demonstrate that TKP-L can enhance the immune capacity of lambs through the microbiota-metabolite axis.

## 5 Conclusions

The above results show that TKP-L can more effectively promote the serum secretion of growth factors such as GH, INS, and IGF-1R, upregulate the levels of serum IgM, IgG, and intestinal IgA antibodies, and stimulate the expression of multiple cytokines than TKP-R. Additionally, TKP-L significantly improves intestinal mucin secretion and gastrointestinal development, boosts the variety and quantity of good bacteria in the gastrointestinal tract, raises the metabolic level of Argininosuccinic acid, and controls several metabolic pathways linked to growth and immunity. In conclusion, TKP-L is more effective than TKP-R in stimulating growth and controlling metabolites and gut microbiota in lambs. Thus, TKP-L can be developed as a natural feed additive to promote lamb growth and modulate the gut microbiota structure.

## Data Availability

The data presented in the study are deposited in the NCBI repository, BioProject ID: PRJNA1434077.

## References

[1] Wang Y Wang R Hao X Hu Y Guo T Zhang J . Growth performance, nutrient digestibility, immune responses and antioxidant status of lambs supplemented with humic acids and fermented wheat bran polysaccharides. Anim Feed Sci Technol. (2020) 269:114644. doi: 10.1016/j.anifeedsci.2020.114644

[2] Stevenson P. Links between industrial livestock production, disease including zoonoses and antimicrobial resistance. Anim Res One Health. (2023) 1:137–44. doi: 10.1002/aro2.19

[3] Su Y Gao X Wang Y Li X Zhang W Zhao J. Astragalus polysaccharide promotes sheep satellite cell differentiation by regulating miR-133a through the MAPK/ERK signaling pathway. Int J Biol Macromol. (2023) 239:124351. doi: 10.1016/j.ijbiomac.2023.124351 37023880

[4] Salehi M Rashidinejad A. Multifaceted roles of plant-derived bioactive polysaccharides: a review of their biological functions, delivery, bioavailability, and applications within the food and pharmaceutical sectors. Int J Biol Macromol. (2025) 290:138855. doi: 10.1016/j.ijbiomac.2024.138855 39701227

[5] Flint HJ Bayer EA Rincon MT Lamed R White BA. Polysaccharide utilization by gut bacteria: potential for new insights from genomic analysis. Nat Rev Microbiol. (2008) 6:121–31. doi: 10.1038/nrmicro1817 18180751

[6] Moheteer A Li J Abulikemu X Lakho SA Meng Y Zhang J . Preparation and activity study of *Ruoqiang jujube* polysaccharide copper chelate. Front Pharmacol. (2024) 14:1347817. doi: 10.3389/fphar.2023.1347817 38273828 PMC10809154

[7] Manafu Z Zhang Z Malajiang X Abula S Guo Q Wu Y . Effects of Alhagi camelorum Fisch polysaccharide from different regions on growth performance and gastrointestinal microbiota of sheep lambs. Front Pharmacol. (2024) 15:1379394. doi: 10.3389/fphar.2024.1379394 38746008 PMC11091474

[8] Wang W Wang Y Cui Z Yang Y An X Qi J. Fermented wheat bran polysaccharides intervention alters rumen bacterial community and promotes rumen development and growth performance in lambs. Front Vet Sci. (2022) 9:841406. doi: 10.3389/fvets.2022.841406 35433917 PMC9007612

[9] Lan J Xu Z Li J Li X Li Y Zhang W. Effects of sea buckthorn polysaccharides on rumen in vitro fermentation characteristics and microbial composition of hu sheep. Microorganisms. (2025) 13:2639. doi: 10.3390/microorganisms13112639 41304322 PMC12654629

[10] Tan M Chu JS Swiger DR. Exploring the medicinal potential of *Taraxacum Kok-Saghyz* (TKS) using widely targeted metabolomics. Metabolites. (2025) 15:306. doi: 10.3390/metabo15050306 40422883 PMC12112865

[11] Wang H Fan X Yu H Li J Cui X Xu X. Characterization of natural rubber concerning its components and molecular weight in *Taraxacum kok-saghyz* rodin using pyrolysis gas chromatography-mass spectrometry. J Sep Sci. (2023) 46:e2201041. doi: 10.1002/jssc.202201041 37609805

[12] Wang S Wu P Fan Z He X Liu J Li M . Dandelion polysaccharide treatment protects against dextran sodium sulfate-induced colitis by suppressing NF-κB/NLRP3 inflammasome-mediated inflammation and activating Nrf2 in mouse colon. Food Sci Nutr. (2023) 11:7271–82. doi: 10.1002/fsn3.3653 37970386 PMC10630811

[13] Talapphet N Palanisamy S Li C Ma N Prabhu NM You S. Polysaccharide extracted from *Taraxacum platycarpum* root exerts immunomodulatory activity via MAPK and NF-κB pathways in RAW264. 7 cells J Ethnopharmacol. (2021) 281:114519. doi: 10.1016/j.jep.2021.114519 34390795

[14] Yu Z Zhao L Zhao JL Xu W Guo Z Zhang AZ . Dietary *Taraxacum mongolicum* polysaccharide ameliorates the growth, immune response, and antioxidant status in association with NF-κB, Nrf2 and TOR in Jian carp (*Cyprinus carpio* var. Jian) Aquaculture. (2022) 547:737522. doi: 10.1016/j.aquaculture.2021.737522

[15] Yan S Dong R. Integrated microbiome-metabolomics analysis reveals the potential mechanism of dandelion root polysaccharides to ameliorate ulcerative colitis. Metabolites. (2024) 14:351. doi: 10.3390/metabo14070351 39057673 PMC11278672

[16] Zhou A Zhang S Yang C Liao N Zhang Y. Dandelion root extracts abolish MAPK pathways to ameliorate experimental mouse ulcerative colitis. Adv Clin Exp Med. (2022) 31:529–38. doi: 10.17219/acem/146234 35178902 PMC13539092

[17] Yan S Yin L Dong R. Inhibition of IEC-6 cell proliferation and the mechanism of ulcerative colitis in C57BL/6 mice by dandelion root polysaccharides. Foods. (2023) 12:3800. doi: 10.3390/foods12203800 37893693 PMC10606498

[18] Bava R Castagna F Lupia C Poerio G Liguori G Lombardi R . Antimicrobial resistance in livestock: a serious threat to public health. Antibiotics (Basel). (2024) 13:551. doi: 10.3390/antibiotics13060551 38927217 PMC11200672

[19] Zhao C Li H Gao C Tian H Guo Y Liu G . Moringa oleifera leaf polysaccharide regulates fecal microbiota and colonic transcriptome in calves. Int J Biol Macromol. 2023;253(Pt 6):127108. doi: 10.1016/j.ijbiomac.2023.127108 37776927

[20] Wang Y Li Z Jin W Mao S. Isolation and Characterization of Ruminal Yeast Strain with Probiotic Potential and Its Effects on Growth Performance, Nutrients Digestibility, Rumen Fermentation and Microbiota of Hu Sheep. J Fungi (Basel). (2022) 8:1260. doi: 10.3390/jof8121260 36547593 PMC9781649

[21] Zhou B Wang H Luo G Niu R Wang J. Effect of dietary yeast chromium and L-carnitine on lipid metabolism of sheep. Biol Trace Elem Res. (2013) 155:221–7. doi: 10.1007/s12011-013-9790-9 23975581

[22] Mihaliková K Gresáková L Boldizárová K Faix S Leng L Kisidayová S. The effects of organic selenium supplementation on the rumen ciliate population in sheep. Folia Microbiol (Praha). (2005) 50:353–6. doi: 10.1007/BF02931418 16408856

[23] Sperling MA. Traditional and novel aspects of the metabolic actions of growth hormone. Growth Horm IGF Res. (2016) 28:69–75. doi: 10.1016/j.ghir.2015.06.005 26194064

[24] Wang LM Feng HL Ma YZ Cang M Li HJ Yan Z . Expression of IGF receptors and its ligands in bovine oocytes and preimplantation embryos. Anim Reprod Sci. (2009) 114:99–108. doi: 10.1016/j.anireprosci.2008.09.019 19013732

[25] Li Z Wang X Wang W An R Wang Y Ren Q . Benefits of tributyrin on growth performance, gastrointestinal tract development, ruminal bacteria and volatile fatty acid formation of weaned small-tailed han lambs. Anim Nutr. (2023) 15:187–96. doi: 10.1016/j.aninu.2023.08.006 38023378 PMC10679854

[26] Ying M Yu Q Zheng B Wang H Wang J Chen S . Cultured Cordyceps sinensis polysaccharides modulate intestinal mucosal immunity and gut microbiota in cyclophosphamide-treated mice. Carbohydr Polym. (2020) 235:115957. doi: 10.1016/j.carbpol.2020.115957 32122493

[27] Cai G Wu C Mao N Song Z Yu L Zhu T . Isolation, purification and characterization of *Pueraria lobata* polysaccharide and its effects on intestinal function in cyclophosphamide-treated mice. Int J Biol Macromol. (2022) 218:356–67. doi: 10.1016/j.ijbiomac.2022.07.153 35878664

[28] Burgueño JF Abreu MT. Epithelial Toll-like receptors and their role in gut homeostasis and disease. Nat Rev Gastroenterol Hepatol. (2020) 17:263–78. doi: 10.1038/s41575-019-0261-4 32103203

[29] Wu X Xu N Ye Z Zhao Q Liu J Li J . Polysaccharide from *Scutellaria barbata* D. don attenuates inflammatory response and microbial dysbiosis in ulcerative colitis mice. Int J Biol Macromol. (2022) 206:1–9. doi: 10.1016/j.ijbiomac.2022.02.119 35218798

[30] Bohländer F Weißmüller S Riehl D Gutscher M Schüttrumpf J Faust S. The functional role of IgA in the IgM/IgA-enriched immunoglobulin preparation trimodulin. Biomedicines. (2021) 9:1828. doi: 10.3390/biomedicines9121828 34944644 PMC8698729

[31] Zuo T Cao L Sun X Li X Wu J Lu S . Dietary squid ink polysaccharide could enhance SIgA secretion in chemotherapeutic mice. Food Funct. (2014) 5:3189–96. doi: 10.1039/C4FO00569D 25308407

[32] Xie SZ Liu B Ye HY Li QM Pan LH Zha XQ . *Dendrobium huoshanense* polysaccharide regionally regulates intestinal mucosal barrier function and intestinal microbiota in mice. Carbohydr Polym. (2019) 206:149–62. doi: 10.1016/j.carbpol.2018.11.002 30553308

[33] Shaikh H Vargas JG Mokhtari Z Jarick KJ Ulbrich M Mosca JP . Mesenteric lymph node transplantation in mice to study immune responses of the gastrointestinal tract. Front Immunol. (2021) 12:689896. doi: 10.3389/fimmu.2021.689896 34381447 PMC8352558

[34] Frederick DR Goggins JA Sabbagh LM Freytag LC Clements JD McLachlan JB. Adjuvant selection regulates gut migration and phenotypic diversity of antigen-specific CD4+ T cells following parenteral immunization. Mucosal Immunol. (2018) 11:549–61. doi: 10.1038/mi.2017.70 28792004 PMC6252260

[35] Forder RE Nattrass GS Geier MS Hughes RJ Hynd PI. Quantitative analyses of genes associated with mucin synthesis of broiler chickens with induced necrotic enteritis. Poult Sci. (2012) 91:1335–41. doi: 10.3382/ps.2011-02062 22582290

[36] Johansson ME Ambort D Pelaseyed T Schütte A Gustafsson JK Ermund A . Composition and functional role of the mucus layers in the intestine. Cell Mol Life Sci. (2011) 68:3635–41. doi: 10.1007/s00018-011-0822-3 21947475 PMC11114784

[37] Jia L Wu J Lei Y Kong F Zhang R Sun J . Oregano essential oils mediated intestinal microbiota and metabolites and improved growth performance and intestinal barrier function in sheep. Front Immunol. (2022) 13:908015. doi: 10.3389/fimmu.2022.908015 35903106 PMC9314563

[38] Yáñez-Ruiz DR Abecia L Newbold CJ. Manipulating rumen microbiome and fermentation through interventions during early life: a review. Front Microbiol. (2015) 6:1133. doi: 10.3389/fmicb.2015.01133 26528276 PMC4604304

[39] Wang L Zhang K Zhang C Feng Y Zhang X Wang X . Dynamics and stabilization of the rumen microbiome in yearling Tibetan sheep. Sci Rep. (2019) 9:19620. doi: 10.1038/s41598-019-56206-3 31873173 PMC6927978

[40] Li C Wang W Liu T Zhang Q Wang G Li F . Effect of early weaning on the intestinal microbiota and expression of genes related to barrier function in lambs. Front Microbiol. (2018) 9:1431. doi: 10.3389/fmicb.2018.01431 30013534 PMC6036172

[41] Zhang Q Zhang T Ta N Zhang J Ding H Zhang X. Replacing dietary alfalfa hay with nettle benefits rumen pH balance and microbiota in rumen and feces with minimal effects on performance and digestibility in dairy cows. Anim Feed Sci Technol. (2024) 307:115825. doi: 10.1016/j.anifeedsci.2023.115825

[42] Pinnell LJ Reyes AA Wolfe CA Weinroth MD Metcalf JL Delmore RJ . Bacteroidetes and Firmicutes drive differing microbial diversity and community composition among micro-environments in the bovine rumen. Front Vet Sci. (2022) 9:897996. doi: 10.3389/fvets.2022.897996 35664853 PMC9161295

[43] Yang H Yang M Fang S Huang X He M Ke S . Evaluating the profound effect of gut microbiome on host appetite in pigs. BMC Microbiol. (2018) 18:215. doi: 10.1186/s12866-018-1364-8 30547751 PMC6295093

[44] Ramayo-Caldas Y Mach N Lepage P Levenez F Denis C Lemonnier G . Phylogenetic network analysis applied to pig gut microbiota identifies an ecosystem structure linked with growth traits. ISME J. (2016) 10:2973–7. doi: 10.1038/ismej.2016.77 27177190 PMC5148198

[45] Schmidt JM Opgenorth L Blaut M. Early-life establishment of the swine gut microbiome and impact on host phenotypes. Environ Microbiol Rep. (2015) 7:418–26. doi: 10.1111/1758-2229.12285 25727666

[46] Duan R Hou J Wang X Huang Z Cao H Hu J . *Prevotella histicola* transplantation ameliorates cognitive impairment and decreases oxidative stress in vascular dementia rats. Brain Sci. (2023) 13:1136. doi: 10.3390/brainsci13081136 37626492 PMC10452631

[47] Mishra A Surolia A. Biochemical characterization of argininosuccinate lyase from M. tuberculosis: significance of a c-terminal cysteine in catalysis and thermal stability. IUBMB Life. (2017) 69:896–907. doi: 10.1002/iub.1683 29044950

[48] Haines RJ Pendleton LC Eichler DC. Argininosuccinate synthase: at the center of arginine metabolism. Int J Biochem Mol Biol. (2011) 2:8–23. PMID: 21494411; PMCID: PMC3074183. 21494411 PMC3074183

[49] Gong J Ma S Xiang H Yang X Zhang W Hu R . Protocatechuic acid attenuated inflammation caused by *Prevotella copri* and its metabolites. Virulence. (2026) 17:2609387. doi: 10.1080/21505594.2025.2609387 41437510 PMC12773465

[50] Fiveable. Argininosuccinate – Biological Chemistry II [Internet]. Editor Becky Bahr. Fiveable. (2024). Available from: https://fiveable.me/key-terms/biological-chemistry-ii/argininosuccinate (Accessed January 25, 2026).

[51] Petrack B Ratner S. Biosynthesis of urea. VII. Reversible formation of argininosuccinic acid. J Biol Chem. (1958) 233:1494–500. PMID: 13610861. doi: 10.1016/S0021-9258(18)49360-X 13610861

[52] Wang S Li X Zhang M Li M. Cetobacterium somerae-derived argininosuccinic acid promotes intestinal and liver ureagenesis to alleviate ammonia intoxication. Microbiome. (2025) 13:163. PMID: 40652219; PMCID: PMC12255148. doi: 10.1186/s40168-025-02152-4 40652219 PMC12255148

[53] Wu G Bazer FW Satterfield MC Gilbreath KR Posey EA Sun Y. L-arginine nutrition and metabolism in ruminants. Adv Exp Med Biol. (2022) 1354:177–206. PMID: 34807443. doi: 10.1007/978-3-030-85686-1_10 34807443

[54] Wu G Meininger CJ McNeal CJ Bazer FW Rhoads JM. Role of L-arginine in nitric oxide synthesis and health in humans. Adv Exp Med Biol. (2021) 1332:167–87. PMID: 34251644. doi: 10.1007/978-3-030-74180-8_10 34251644

