# Correction: Mechanism underlying the regulation of gut microbiota-metabolite axis and growth/immune function in lambs by leaf-derived polysaccharides from *Taraxacum kok-saghyz*

**DOI:** 10.3389/fvets.2026.1853032

**Published:** 2026-08-07

**Authors:** Alimu Aersilan, Zulikeyan Manafu, Gulibanu Aosiman, Xiaohui Zhang, Ziluola Tuxunjiang, Yan Meng, Saifuding Abula, Adelijiang Wusiman

**Affiliations:** 1 College of Veterinary Medicine, Xinjiang Agricultural University, Urumqi, China; 2 Xinjiang Key Laboratory of New Drug Study and Creation for Herbivorous Animal (XJ-KLNDSCHA), Xinjiang Agricultural University, Urumqi, China; 3 Crop Research Institute of Xinjiang Uygur Autonomous Region Academy of Agricultural Sciences/National Central Asian Characteristic Crop Germplasm Resources Medium-Term Gene Bank, Urumqi, China

**Keywords:** Gastrointestinal microorganisms and metabolism, growth efficiency, intestinal immunity, sheep lambs, *Taraxacum kok-saghyz* polysaccharide

An incorrect Funding statement was provided in the published article. The correct funder is “The Project for Supporting Xinjiang Uygur Autonomous Region Frontier Science and Technology Support Program for Talent (Grant No. XJRC-2025KJKJQY002).

Funder “Tianshan Talent” Leading Scientific and Technological Innovation Program (2023TSYCLJ0010), and Xinjiang Uygur Autonomous Region Talent Development Fund were erroneously omitted.

The corrected funding statement appears below:

## Funding

The author(s) declared that financial support was received for this work and/or its publication. The author declares that his research, writing, and article publication have all received financial support. The Project for Supporting Xinjiang Uygur Autonomous Region Frontier Science and Technology Support Program for Talent (Grant No. XJRC-2025KJKJQY002); Xinjiang Uygur Autonomous Region “Tianshan Talent” Leading Scientific and Technological Innovation Program (2023TSYCLJ0010); The second round of support funds for the Xinjiang Uygur Autonomous Region Talent Development Fund in 2025-Tianchi Talent Introduction Program.

The original version of this article has been updated.

